# Buildings Contemplated and in Progress

**Published:** 1913-10-04

**Authors:** 


### Buildings Contemplated and in Progress.

Ashton-under-Lyne.—The Governors of the District Infirmary have under consideration a scheme for extension and improvements estimated to cost from £5,000 to £6,000. Messrs. W. H. George and Son are the architects to the infirmary.

Barmouth.—A site near Barmouth for a sanatorium for Merionethshire has been purchased by the Committee of the Welsh National Memorial to King Edward VII., and it has been decided to proceed with the building at once.

Barnsley.—The Town Council has decided to apply to the Local Government Board for sanction to borrow £2,200 for the purchase and alteration of premises in Queen's Road for a tuberculosis dispensary.

Basford.—The Guardians have been authorised to borrow £2,800 to cover the cost of erecting a nurses' home.

Bedwellty.—Instructions have been issued by the Guardians for the preparation of plans for the extension of the school block at the workhouse to provide sleeping accommodation for the nurses.

Bideford.—The Devon County Council propose to erect a tuberculosis hospital at Bideford, and is negotiating for a site.

Brackley.—Plans and an estimate at £200 for alterations to the workhouse are being considered by the Guardians.

Bridgwater.—It is proposed to extend the Bridgwater Hospital at a cost of £5,000. The architects to the hospital are Messrs. Samson and Colthurst.

Bridport.—The design of Messrs. F. H. Shayler and E. R. Bill, Wrekin Hotel Chambers, Wellington, Salop, has been placed first by the assessors, Messrs. Wheeler and Searle, of the plans submitted in competition for the Bridport Cottage Hospital.

Coxlodge.—Mr. Dyson, of Newcastle, is architect for the extension of the Newcastle-on-Tyne Asylum, Coxlodge, and the contractor is Mr. Pringle, of Gateshead. The cost of the scheme is estimated at £21,000.

Crediton.—The Devon County Council propose to provide the Crediton Anti-Consumption Association with a pavilion and shelters at a cost of £100.

Darlington.—Plans submitted by the Guardians for providing accommodation at the workhouse for short-term lunatics have been approved by the Local Government Board. The cost is estimated at £650.

Evesham.—Plans for a tuberculosis pavilion and additions to the administrative block at Evesham Sanatorium have been passed by the Evesham Joint Hospital Board.

Fulwood.—Plans submitted by the Preston Guardians for a nurses' home at the Fulwood Workhouse have been approved by the Guardians. The cost is estimated at £4,800. Mr. F. Howorth, 6 Cannon Street, Preston, is the architect.

Hill Top.—The Worcestershire County Council has applied to the Local Government Board for sanction to borrow £800 towards the cost of erecting an additional pavilion at the hospital at Hill Top, Bromsgrove.

Howdon.—The Rural District Council has resolved to apply to the Local Government Board for sanction to borrow £360 for a site for an isolation hospital.

Normanton.—The Normanton and District Joint Isolation Hospital Committee has decided to obtain tenders for additions to the building, the cost of which is estimated at £5,000.

St. Austell.—The Guardians have under consideration plans prepared by the architect, Mr. B. C. Andrew, for alterations to the workhouse infirmary.

Sherborne.—The Urban District Council has issued instructions for the preparation of plans for rebuilding the isolation hospital.

Summerfield.—The Aberdeen District Committee are considering plans prepared by Messrs. Jenkins and Marr, of Aberdeen, for the extension of a pavilion at Summerfield Hospital, the cost of which is estimated at £1,425.

Wolsingham.—The Durham County Council has received the approval of the Local Government Board to the purchase of the Holywood Hall estate at Wolsingham as a site for a sanatorium.

